# QSAR Study of 17β-HSD3 Inhibitors by Genetic Algorithm-Support Vector Machine as a Target Receptor for the Treatment of Prostate Cancer

**Published:** 2017

**Authors:** Eslam Pourbasheer, Saadat Vahdani, Davood Malekzadeh, Reza Aalizadeh, Amin Ebadi

**Affiliations:** a *Department of Chemistry, Payame Noor University Tehran, Iran.*; b *Department of Chemistry, Islamic Azad University-North Tehran Branch, Tehran, Iran. *; c *Laboratory of Analytical Chemistry, Department of Chemistry, National and Kapodistrian University of Athens, Panepistimiopolis Zografou, 15771 Athens, Greece.*; d *Department of Chemistry, Kazerun Branch, Islamic Azad University, Kazerun, Iran.*

**Keywords:** QSAR, Genetic algorithms, Support vector machine, Multiple linear regressions, 17β-HSD3

## Abstract

The 17β-HSD3 enzyme plays a key role in treatment of prostate cancer and small inhibitors can be used to efficiently target it. In the present study, the multiple linear regression (MLR), and support vector machine (SVM) methods were used to interpret the chemical structural functionality against the inhibition activity of some 17β-HSD3inhibitors. Chemical structural information were described through various types of molecular descriptors and genetic algorithm (GA) was applied to decrease the complexity of inhibition pathway to a few relevant molecular descriptors. Non-linear method (GA-SVM) showed to be better than the linear (GA-MLR) method in terms of the internal and the external prediction accuracy. The SVM model, with high statistical significance (R^2^_train _= 0.938; R^2^_test _= 0.870), was found to be useful for estimating the inhibition activity of 17β-HSD3 inhibitors. The models were validated rigorously through leave-one-out cross-validation and several compounds as external test set. Furthermore, the external predictive power of the proposed model was examined by considering modified R^2^ and concordance correlation coefficient values, Golbraikh and Tropsha acceptable model criteriaʹs, and an extra evaluation set from an external data set. Applicability domain of the linear model was carefully defined using Williams plot. Moreover, Euclidean based applicability domain was applied to define the chemical structural diversity of the evaluation set and training set.

## Introduction

There are growing concerns over treatment of prostate cancer as the death ratio increases, and it requires of an emerging need for development of new and efficient drugs (1, 2). The treatment therapy for such disease is of great discussions as they are frequently failing or are selective for patients. There are still some rooms to discuss for an optimal therapy for a progressive prostate cancer. Prostate cancer is an androgen sensitive disease in which androgens testosterone (T) and dihydro testosterone (DHT) play major roles in development of this type of cancer (1, 2). Currently, an efficient therapy is to manage the prostate cancer either from the central regulation of androgen biosynthesis or by blocking of androgen receptor (3). The production of androgens is restricted to two steps within the central nervous system; it is also locally controlled in peripheral organs that are targeted. The active androgens T and DHT are directly synthesized by conversion of the inactive forerunner and rostenedione (Δ4-dione) in presence of 17β-hydroxysteroid dehydrogenases (17β-HSDs) (3). In fact, the 17β-HSDs arbitrate the last step in the alteration of sex steroids in the peripheral target tissues (3, 4). The final step in the biosynthesis of a potent androgen T is controlled by 17β-HSD3 and NADPH as a cofactor through reduction of the C17 ketone of Δ4-dione (5). Since 17β-HSD3 is largely uttered in testes and prostate tissue at some prostate tumors, it is believed to play crucial role in both gonadal and non-gonadal T biosynthesis (1, 4). In this regard, this enzyme is such an attractive target of small inhibitors for the treatment of prostate cancer (3, 6-8).

Many medicines are typically developed using several blind trials which could be costly, time-consuming or failed to show high inhibition. Using theoretical methods as alternative tools for predicting activities of chemicals could help decreasing the chance of having false negatives prior to any trials. Among the theoretical methods, the quantitative structure-activity relationships (QSAR) have been successfully established to predict different important biopharmaceutical properties, including genotoxicity, toxicity, oral bioavailability, carcinogenicity, and mutagenicity (9-11). There are plenty of literatures reporting the application of computational methods for describing the bioactivities of newly synthesized compounds (12-15). The main aim of QSAR studies is to establish an empirical rule or a function to correlate set of chemical descriptors of compounds to their bioactivities. Different modeling techniques thus can be critical to achieve a good QSAR model. Multivariate modeling techniques have been widely employed in QSAR studies such as multiple linear regressions (MLR) (16-21), partial least squares analysis (PLS) (22), principal component regression (PCR) (23), artificial neural networks (ANN) (24, 25), and support vector machine (SVM) (11, 26). Such methods require a few numbers of relevant molecular features for the sake of simplicity in interpreting the mechanism of act and also preventing over-fitting. Based on probabilistic choice, genetic algorithm (GA) offers high capability to select set of molecular features having the best fit to explain the existed problem (27, 28).

In this work, a QSAR based approach was conducted to interpret the chemical functionality toward 17β-HSD3 inhibition activity for set of potent inhibitors. GA-MLR and GA-SVM were two modelling techniques used to develop these QSAR models for 17β-HSD3 inhibitors. Various validation protocols were used to investigate the accuracy of proposed models and finally, some new compounds were designed and their activities were predicted. The results derived from GA-SVM method were compared to the results of GA-MLR method and showed to be more accurate

## Experimental

*Data set*

A data set consists of 35 compounds as 17β-HSD3 inhibitors for the QSAR workflow was adopted from the literature (3) as listed in Table 1. The pIC_50 _= −log [IC_50_ (M)] values were used as the dependent variables so as to give numerically larger values. The chemical structures and corresponding pIC_50_ values are shown in Table 1.

*Descriptor calculations and reduction*

The 2D structures of the molecules were drawn using HyperChem 7 software (29). For these molecules, the conformers having less energy were obtained using the semi empirical AM1 method. The molecular structures were optimized using the Polak-Ribiere algorithm until the root mean square gradient reaches 0.01 kcal/mol. These conformers were sent to Dragon program (30) to calculate molecular descriptors of 0D (Constitutional), 1D (Functional group and Atom-Centred Fragment, Empirical and Properties descriptors), 2D (Topological, Molecular Walk Count, BCUT, Galvez topol. Charge indices and 2D autocorrelations descriptors) and 3D classes [Charge, aromaticity indices, Randic molecular profile, Geometrical, Radial Distribution Function, 3D-MoRSE (3Dmolecular Representation of Structure based on Electron diffraction), WHIM (Weighted Holistic Invariant Molecular descriptors), GETAWAY (Geometry, Topology and Atoms- Weighted AssemblY)], creating total of 1497 descriptors. The calculated descriptors were screened for the existence of constant or near constant variables and removed from the data matrix. In addition, the correlation between descriptors as well as the activity was examined and the collinear descriptors (*i.e.* r > 0.9) were detected. Among the collinear descriptors, the one presenting the highest correlation with the activity was retained and the others were removed from the data matrix. After these steps, the number of descriptors was reduced to 519.

*Clustering*

**Figure 1:**
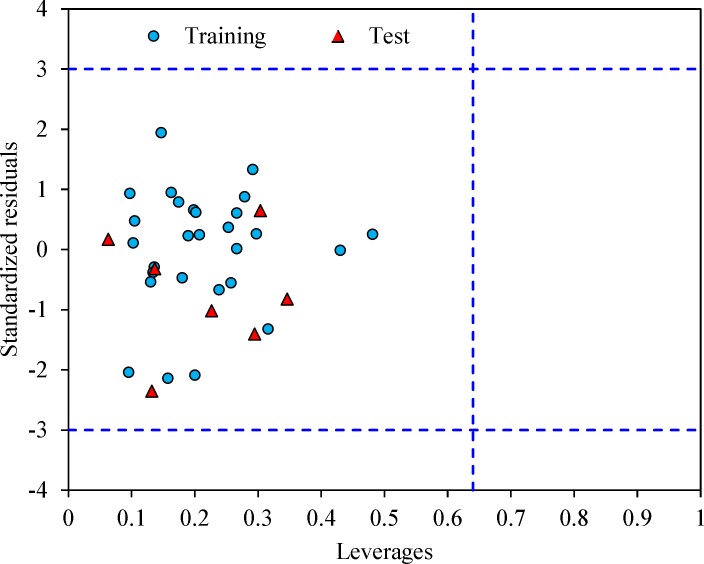
A dendrogram illustrating the results of the hierarchical clustering of the training and test sets

One of the most important steps in a QSAR study is to divide the data set into training and test sets correctly to avoid any information lost during creation of models and fitting step. Here, a hierarchical cluster method was used while comparing the diversity of chemical structure and their related activities. Hierarchical cluster method is a statistical approach for finding relatively homogeneous clusters of cases based on measured characteristics (31, 32). It starts with each case in a separated cluster, and then, combines the clusters sequentially, reducing the number of clusters at each step until only one cluster is left. When there are N cases, this involves N-1 clustering steps, or fusions. This hierarchical clustering process can be represented as a tree or a dendrogram that each step in the clustering process is illustrated by a linkage. The selection of the training and test sets was done in a random way from each cluster with close investigation of the activity of each selection. The justification behind the selection can be defined in two steps; a) the range of the activity values of both the training set and the test set should be covered from the lowest to the highest; b) each selected data point for the test set should show high distance linkage in the dendrogram from the previously chosen one. Consequently, a training set of 28 compounds to develop the model and a test set of 7 compounds to evaluate the model were comprised. The dendrogram of used data set is shown in Figure 1.

## Results and Discussion

The prediction ability of QSAR models is affected by two factors. One is the descriptors, which must carry enough information of molecular structures for interpreting how the observed activity correlates to chemical structure; the other one is the modeling method employed. There are pools of descriptors which may cause over fitting of statistical methods. Therefore, identifying important descriptors certainly is a need in QSAR studies. In this study, the genetic algorithm coded in the MATALAB software was used to select relevant descriptors for building the QSAR models. For the selection of the most important descriptors, GA ran many times with different initial sets of population creating different final models. Among these models, one model presented the highest statistical quality was selected and reported. Five descriptors were selected by this method and used to construct linear and nonlinear models based on MLR and SVM techniques. 

*MLR analysis*

In order to build and test the linear model a data set of 35 compounds was divided by hierarchical cluster method into a training set of 28 compounds, which was used to build the model, and into a test set of 7 compounds which was applied to evaluate it. The following equation based on five molecular descriptors is obtained:

pIC_50 _= 11.510 (± 1.329) + 249.056 (± 45.566) GATS6m - 2.156 (± 0.826) GATS1e + 4.497 

(± 1.624) P2e - 33.803 (± 5.075) R7u^+^ -0.95 (± 0.232) C-026                     (1)

**Figure 2:**
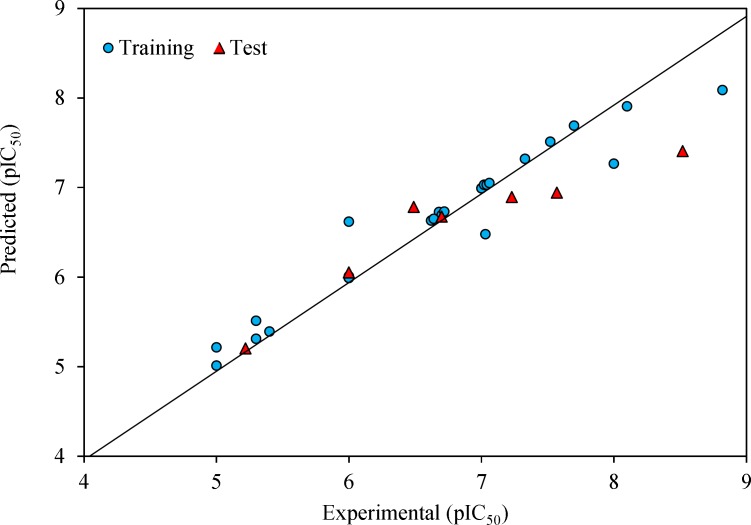
Predicted versus experimental pIC_50_ by GA-MLR model

The prediction results are given in Table 1 and shown in Figure 2. The square correlation coefficient (R^2^) and Fisher F statistic (F) are 0.779 and 15.508 for the training set, respectively. The root mean square error value (RMSE = 0.443) is lower enough to indicate successful predictions of the QSAR model developed by GA-MLR. Then, the built model was used to predict the test set data. The statistical external validation (R^2^_Test _= 0.823, F_Test _= 0.675, RMSE_Test _= 0.531) confirmed the high satisfactory prediction ability for the compounds that were not used during the model development.

The model obtained was internally validated using leave-one-out (LOO) cross-validation process. For LOO cross-validation, a compound was removed from the set, and the model was recalculated. The predicted activity for that compound was then compared to its actual value. This is repeated until all compounds omitted once. High value for correlation coefficient of cross-validation (Q^2 ^= 0.674) indicates that the obtained regression model has a good internal predictive power. In addition, the robustness of the proposed model and its predictive ability was guaranteed by a high Q^2^_BOOT_ (Q^2^_BOOT _= 0.665) based on bootstrapping repeated 5000 times. This indicates that the proposed regression model has a good internal predictive power.

**Table 1:** Experimental and predicted pIC_50_ values for 17β-HSD3 inhibitors using GA-MLR and GA-SVM models

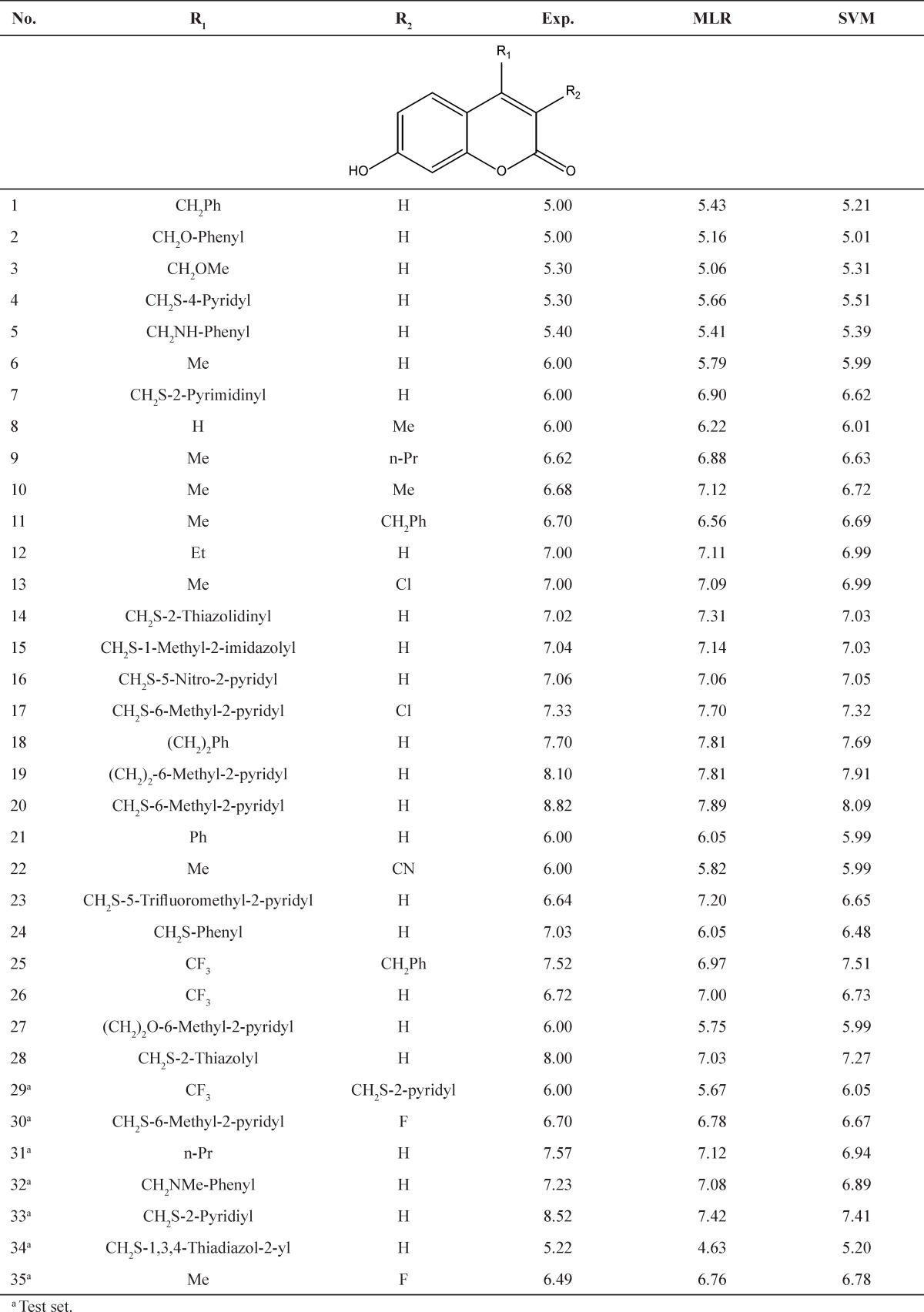

a Test set.

**Table 2.** The Q^2^_LOO_ and R^2^_training_ values after several Y-randomization tests

**No.**	**Q** ^2^	**R** ^2^
1	0.114	0.036
2	0.058	0.103
3	0.058	0.137
4	0.139	0.048
5	0.091	0.104
6	0.046	0.287
7	0.006	0.180
8	0.001	0.244
9	0.452	0.078
10	0.003	0.169

**Table 3:** The correlation coefficient of selected descriptors and corresponding VIF values by GA-MLR.

	**GATS6m**	**GATS1e**	**P2e**	**R7u+**	**C-026**	**VIF** a
GATS6m	1	0	0	0	0	1.047
GATS1e	0.095	1	0	0	0	1.172
P2e	-0.080	0.297	1	0	0	1.495
R7u+	0.078	0.255	0.503	1	0	1.441
C-026	0.209	-0.105	-0.217	-0.220	1	1.052

a Variation inflation factor.

**Table 4:** The statistical parameters of different constructed QSAR models

	**Training**						
	R^2^	RMSE			F	CCC	R^2^_adj_
GA-MLR	0.779	0.444			15.508	0.8758	0.729
GA-SVM	0.938	0.260			42.831	0.9563	0.924
	**Test**						
	R^2^		RMSE	F		CCC	rm^2^
GA-MLR	0.823		0.531	0.675		0.8554	0.775
GA-SVM	0.870		0.513	0.390		0.8257	0.667

CCC: concordance correlation coefficient.

**Table 5:** Experimental validation of models based on evaluation external set.

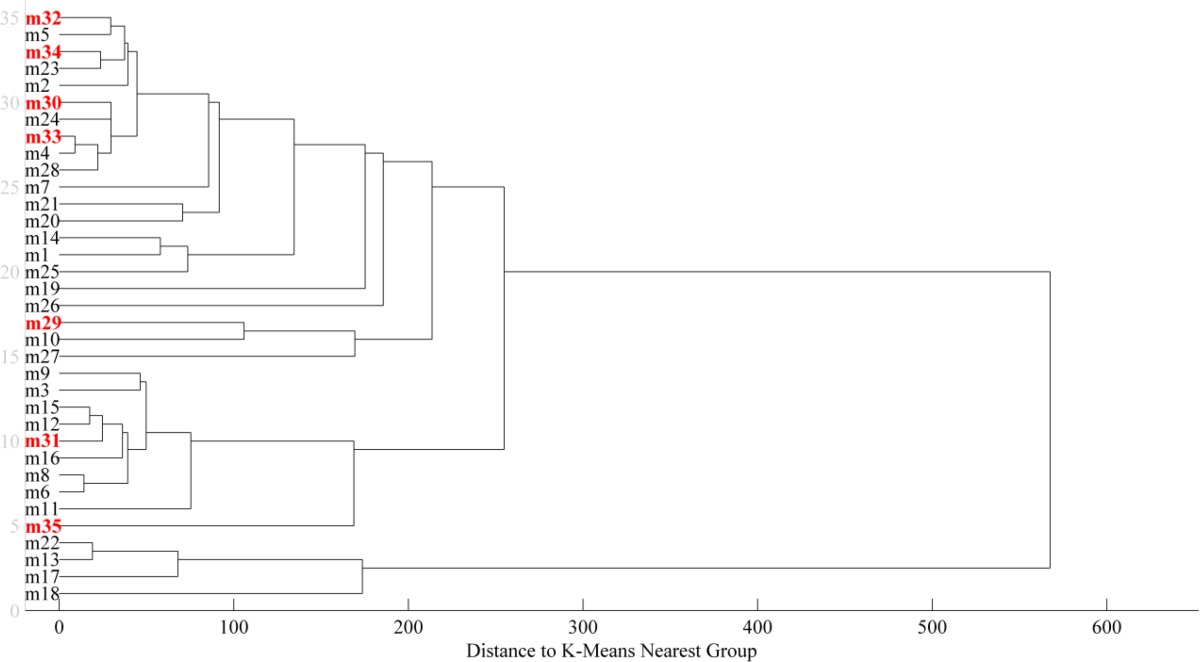

a See reference (42).

b See reference (3).

c See reference (8).

d Based on Euclidean applicability domain, the molecules are within applicability domain of models.

**Table 6:** Golbraikh and Tropsha acceptable model criteria's for GA-MLR.

	**Values for GA-MLR**	**GA-MLR**
Condition I	0.674	Passed
Condition II	0.823	Passed
Condition III	K = 1.049 K′= 0.950 $R^{2}-{R_{0}}^{2}/R^{2}$ = 0.004 ${R_{0}}^{2}-{R_{0}}^{\mathrm{'2}}/R^{2}$ = 0.020	Passed
Condition IV	${R_{0}}^{2}-{R_{0}}^{\mathrm{'2}}$ = 0.0123	Passed

**Table 7:** Statistical parameters comparison based on different selected descriptors by GA-MLR

**Linear model equation**							
Model 1:	pIC_50_ = 7.0086 (± 0.44447) - 0.56701 (± 0.28122) nN + 0.21651 (± 0.0772) RDF100u + 0.22206 (± 0.05459) RDF070e - 0.29424 (± 0.11456) RDF065p - 1.16837 (± 0.40968) Mor15m						
Model 2:	pIC_50_= 6.09379 (± 0.76707) - 0.4853 (± 0.28032) nN + 0.46193 (± 0.33757) GATS6e + 0.19134 (± 0.04665) RDF070e - 1.19599 (± 0.38172) Mor10m - 1.03101 (± 0.39102) Mor15m						
Model 3:	pIC_50 _ = 6.72661 (± 0.47912) + 0.23447 (± 0.34966) nBnz - 0.05179 (± 0.01078) Eig1p + 0.24754 (± 0.049) RDF070e - 0.43443 (± 0.14922) Mor03m + 1.79601(± 0.46346) C - 029						
Model 4 :	pIC_50_ = 6.48189 (± 0.61284) + 0.20329 (± 0.3866) nBnz - 0.0438 (±0.01101) Eig1p + 0.255 (± 0.05382) RDF070e + 0.39064 (± 0.2075) H0m + 1.71489 (± 0.50505) C-029						
Model 5:	pIC_50_= 6.61855 (± 0.58588) + 0.1342 (± 0.38201) nBnz - 0.04662 (± 0.01173) Eig1p + 0.2437 (± 0.05394) RDF070e + 0.12274 (± 0.06922) RTm + 1.71805 (±0.50909) C-029						
Model 6:	pIC_50_= 6.9527 (± 0.42856) 0.69797 (± 0.28258) nN + 0.18259 (± 0.04581)RD F070e - 1.11912 (± 0.35743) Mor10m - 1.06653 (± 0.37879) Mor15m + 0.53212 (± 0.31188) C-029						
Model 7:	pIC_50_ = 7.4772 (± 0.43517) - 0.59149 (± 0.25509) nN + 0.21837 (± 0.06629) RDF100u - 0.1982 (± 0.05626) RDF065m + 0.20205 (± 0.04439) RDF070e - 1.67878 (± 0.36124) Mor15m						
Model 8:	pIC_50_ = 6.8958 (± 0.43333) + 0.01576 (± 0.22858) nN - 0.04743 (± 0.00872) Eig1p + 0.23367 (± 0.04769) RDF070e - 0.40774 (± 0.15963) Mor03m + 1.60567 (± 0.37707) C-029						
Model 9:	pIC_50_ = 6.65879 (± 1.20624) + 0.08351 (± 0.4122) IDDE - 0.04773 (± 0.00881) Eig1p + 0.23025 (± 0.05042) RDF070e - 0.40408 (± 0.15195) Mor03m + 1.60321 (± 0.37471) C-029						
Model 10:	pIC_50_ = 6.77014 (± 0.44483) - 0.56233 (± 0.28016) nN + 0.11016 (± 0.04713) RDF070e - 1.2491 (± 0.38605) Mor15m - 0.95591(± 0.35215) Mor10e + 0.60422 (± 0.33097) C-029						
Statistical Results							
			** R** ^2^_train_	** F**_train_	** Q** ^2^_LOO _	** R** ^2^_test_	**rm** ^2^_test_
Model 1			0.608	6.820	0.333	0.736	0.702
Model 2			0.618	7.081	0.389	0.714	0.498
Model 3			0.692	9.856	0.452	0.820	0.771
Model 4			0.632	7.549	0.420	0.878	0.704
Model 5			0.626	7.362	0.409	0.836	0.718
Model 6			0.633	7.581	0.360	0.766	0.616
Model 7			0.674	9.101	0.399	0.790	0.613
Model 8			0.685	9.574	0.446	0.811	0.722
Model 9			0.686	9.597	0.464	0.803	0.720
Model 10			0.602	6.663	0.370	0.805	0.584
*Main model*			*0.779*	*15.50*	*0.674*	*0.823*	*0.775*
nN:		Number of Nitrogen atoms					
RDF100u:		Radial Distribution Function - 100 /unweighted					
RDF070e:		Radial Distribution Function - 070 / weighted by Sanderson electronegativity					
RDF065p:		Radial Distribution Function - 065 /weighted by polarizability					
Mor15m:		Signal 15 / weighted by mass					
GATS6e:		Geary autocorrelation of lag 6 weighted by Sanderson electronegativity					
Mor10m:		Signal 10 / weighted by mass					
nBnz:		Number of benzene-like rings					
Eig1p:		Leading eigenvalue from polarizability weighted distance matrix					
Mor03m:		Signal 03 / weighted by mass					
C-029:		R-CX-X					
H0m:		H autocorrelation of lag 0 /weighted by mass					
RTm:		R total index / weighted by mass					
RDF065m:		Radial Distribution Function - 065 /weightedby mass					
IDDE:		Mean information content on the distance degree equality					
Mor10e:		Signal 10 / weighted by Sanderson electronegativity					

**Table 8:** Design of some novel inhibitors with the predicted inhibition activities.

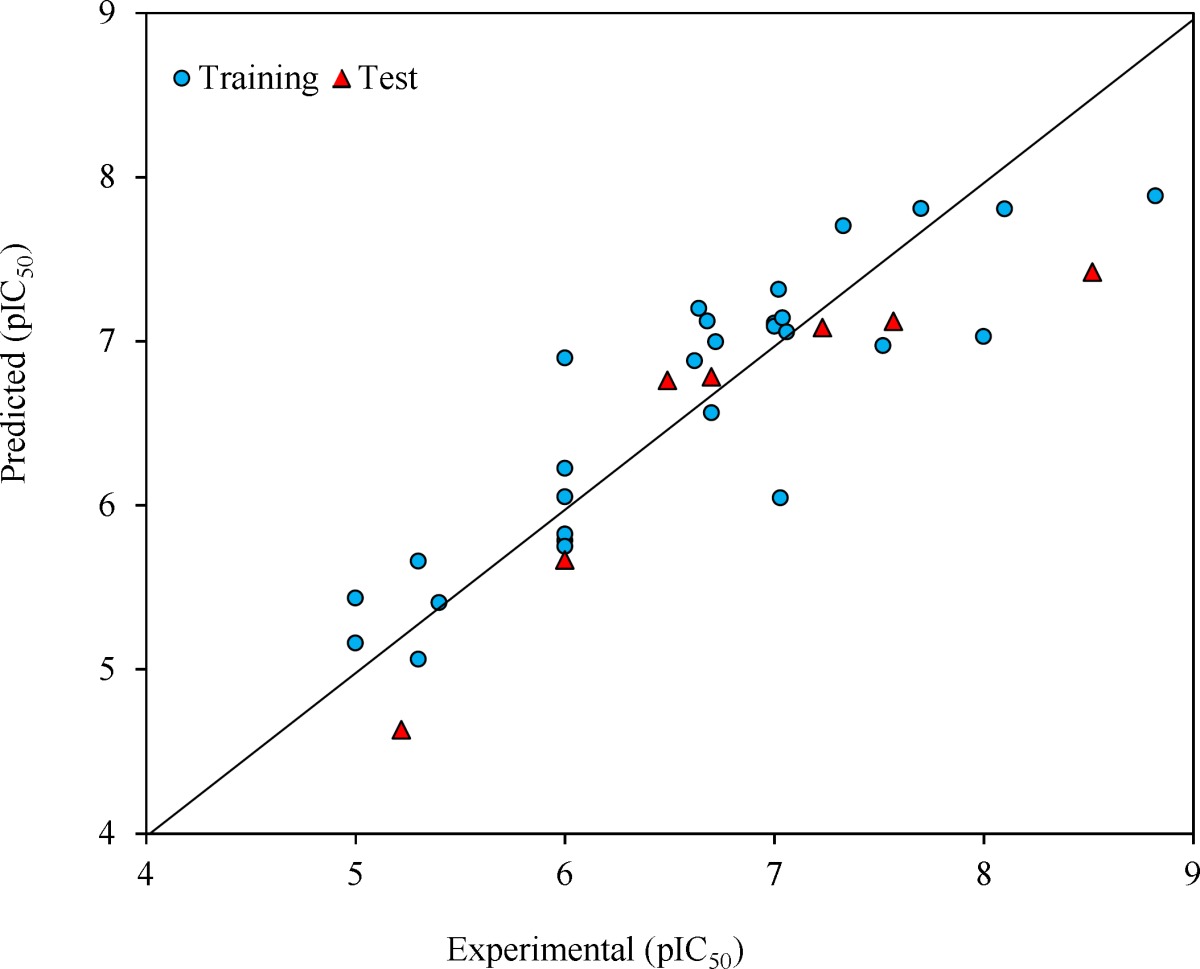

**Figure 3:**
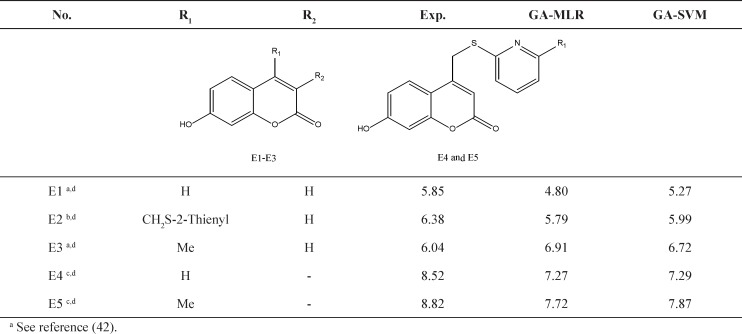
The Williams plot of the training and test sets

**Figure 4.**
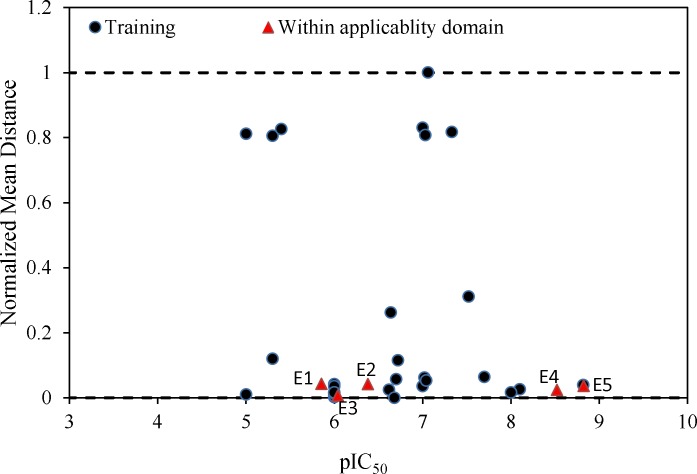
Predicted versus experimental pIC_50_ by GA-SVM model

**Figure 5:**
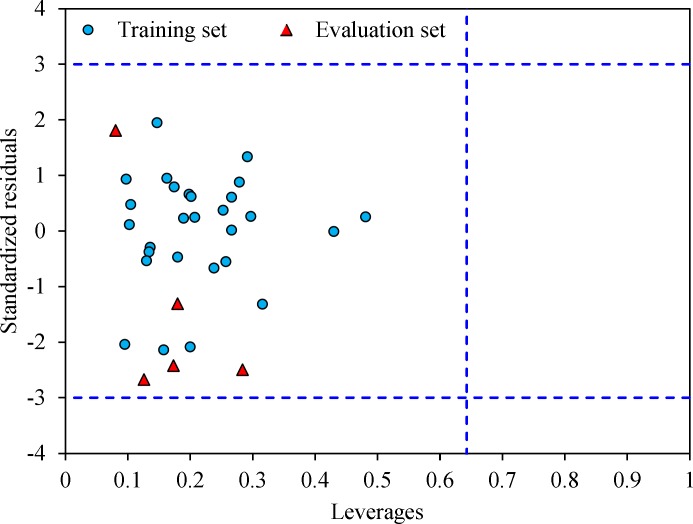
Euclidean based applicability domain of the proposed models

**Figure 6:**
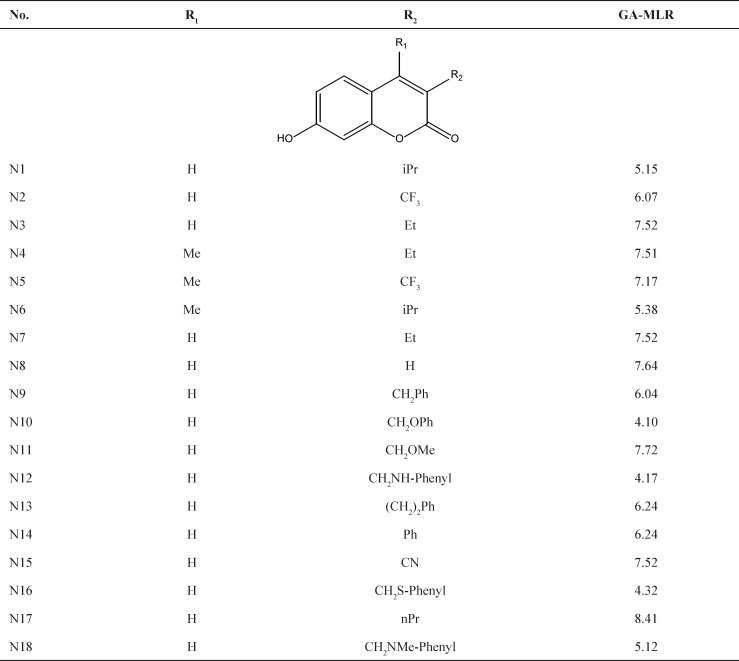
The Williams plot of the training and evaluation sets

Y-randomization test is a widely used technique to evaluate the robustness of QSAR model in terms of correlation obtained by chance. In this technique, the dependent variable (Y vector) randomly shuffles, and new QSAR models develop. This process gets repeated for 10 times. The lower values of R^2^ and Q^2^ in the shuffled cases via Y-randomization test confirm the robustness of the QSAR model (33). The statistical data of R^2^ and Q^2^ for all runs are listed in Table 2.

The leverage values can be calculated for every compound and plotted versus standardized residuals, and it allows a graphical identification of both outliers and the influential chemicals in a model. Figure 3 shows the Williams plot. The applicability domain is established inside a squared area within ± 3 bound for residuals and a leverage threshold h* (h* = , where is the number of model parameters plus one and n is the number of compounds) (34). It shows that all compounds for the training set and test set are inside of this area. Figure 3 indicates that that there are obviously no compounds with standard residuals > 3δ for both the training and test sets (outlier). All the chemicals have the leverage less than the warning h* value of 0.64.

*Interpretation of descriptors*

By interpreting the descriptors introduced by QSAR model, it is possible to gain some insights into chemical features that reveal the contributions of functional group toward the inhibition activity of the 17β-HSD3 inhibitors. The first and second descriptors in the proposed model are GATS6m and GATS1e. These descriptors belong to the 2D autocorrelation indices descriptors. For these descriptors, the Geary coefficient is a distance-type function that can be any physico-chemical property calculated for each atom, such as atomic mass, polarizability, *etc.* Therefore, the atoms represent the set of discrete points in space and the atomic property is the function evaluated at those points. GATS6m is the mean Geary autocorrelation - lag 6 /weighted by atomic masses. The physico-chemical property in this case is atomic mass. GATS6m descriptor displays a positive coefficient in equation 1 which indicates that the pIC_50_ value directly relates to this descriptor. Hence, it is concluded that by increasing the atomic masses, the value of this descriptor increasing, cause an increase in its pIC_50_ value. GATS1e is the Geary autocorrelation lag 1/weighted by atomic Sanderson electronegativities containing information about atomic electronegativities. In this case, the path connecting a pair of atoms has length 1 and involves the atomic Sanderson electronegativities as weighting scheme to distinguish their nature. This descriptor displays a negative sign, which indicates that the pIC_50_ is inversely related to the atomic electronegativities.

The third descriptor is P2e (second component shape directional WHIM index weighted by atomic Sanderson electronegativities). It is one of the WHIM descriptors which are based on the statistical indices calculated from the projections of atoms along principal axes. The algorithm consists of performing a principal components analysis of the centered Cartesian coordinates of a molecule by using a weighted covariance matrix obtained from different weighing schemes for the atoms. The atomic Sanderson electronegativity is one of the weighting schemes that is used for computing the weighted covariance matrix in this descriptor (P2e). The P2e has a positive sign which indicates that pIC50 directly relates to this descriptor; therefore, increasing the value of this descriptor for a molecule leads to increase in its pIC_50_ value.

The forth descriptor is R7u+ (R maximal autocorrelation of lag 7/unweighted). It is one of the GETAWAY descriptors. GETAWAY descriptors encode both the geometrical information given by the inﬂuence molecular matrix and the topological information derived from the molecular graph. The weighting function is any physicochemical properties in selected atoms (26). The negative sign of this descriptor indicates that the pIC_50_ inversely relates to R7u value.

The C-026 descriptor belongs to atom-centred fragments. This provides information about the number of predefined structural features in the molecule, which in this case is R–CX–R. The C-026 displays a negative sign indicating that the pIC_50_ inversely relates to the C-026 descriptor. It was concluded that by increasing the number of R-CX-R substations of molecules the pIC_50_ value would decrease. Multi-collinearities for the above descriptors were inspected by calculating their variation inflation factors (VIF) as follows:

$\mathrm{VIF}=\frac{1}{1-R^{2}}$

 (2)

Where r in the formula is; the correlation coefficient of multiple regression between a variable and the others in the model (35). Correlation coefficient and corresponding VIF values for each descriptor are given in Table 3. All correlation coefficient values were less than 0.51 indicating that the selected descriptors are independent. All variables have VIF less than 5 indicating that the selected descriptors are not highly correlated and the developed model has high statistical significance (35).

*Support vector machine*

In addition to linear model, the non-linear model was also built by support vector machine based on the same subset of descriptors used in GA-MLR model. The SVM method originally proposed and developed by Vapnik (36). Its main advantage is adopting the structure risk minimization (SRM) principle, minimizing an upper bound of the generalization error on the Vapnik-Chernoverkis dimension. This is shown to be superior to a traditional empirical risk minimization (ERM). More details about SVM can be found in our previous works (11, 26, 37).

RMSE of leave one out cross-validation was used as fitness function to optimize SVM model. Performance of SVM for regression purpose depends on the combination of several factors like kernel function type, capacity parameter C, ε of ε-insensitive loss function, and Gamma (γ).

First, the kernel function should be determined, which represents the sample distribution in the mapping space. In this work, the radial basis function (RBF) was used because it offered a good general performance. The next step in the construction of SVM model was optimizing its parameters, including γ, ε-insensitive and C. The optimization of SVM parameters was performed by changing their values in the training step and calculating the RMSE of leave one out cross-validation for the model. 

γ as the kernel function affects the number of support vectors, which has a close relation with the performance of the SVM and training time. Also, the γ controls the amplitude of the RBF function and, therefore, controls the generalization ability of SVM. The optimal value of γ was obtained at 5.

The optimal value for ε depends on the type of noise presented in the data, which is usually unknown. ε -insensitivity prevents the entire training set to meet boundary conditions and allows the possibility of sparsity in the dual formulations solution. Thus, choosing the appropriate value of ε is a critical step. The ε = 0.01 was selected as optimal value. The other parameter is a regularization parameter C that controls the trade-off between maximizing the margin and minimizing the training error. If C is too small, then insufficient stress will be placed on fitting the training data. On the other hand, if C is too large, then the SVM model will over-fit on the training data. The C = 50 was selected as the optimal value. 

After optimizing SVM parameters, it was used to predict the pIC_50_ of training and test sets. The statistical parameters of this model are R^2 ^= 0.938, and RMSE = 0.260 for the training set, and R^2 ^= 0.870, and RMSE = 0.513 for the test set. The predicted against the experiment pIC_50_ values by GA–SVM method are plotted in Figure 4 and also are shown in Table 1.

The comparison results of the proposed models by SVM and MLR are presented in Table 4. As can be seen, the RMSE of SVM method has less value for the training and test sets than the MLR method. Low RMSEs indicates more accurate model. In addition to above statistical parameters, external predictive power of both proposed models using a test set was examined by considering modified R^2^ (38) and to further investigate the accuracy and precise of a model, concordance correlation coefficient method can be used (39). Concordance correlation coefficient (CCC) evaluates the degree to which pairs of observations fall in the 45° line through the origin. These results show the superiority of GA-SVM model against the GA-MLR model.

*Experimental and Theoretical Validation*

Further validation protocols were followed to be sure that these models are applicable for prediction of the subsequent novel molecules. Since the developed models met the initial acceptance criteria, the Golbraikh and Tropsha acceptable model criteriaʹs was also followed (40). As discussed, refereeing to Q^2^_LOO _and R^2^ values for presenting the predictive ability of a built model is not sufficient in all cases, and it was claimed that the predictive power of a model can be investigated only based on the test set compounds. Therefore, a true and valid model can be established only on the biases of model validation procedure consisted of prediction of activities or properties of compounds not included in the model structure. Despite the generation of different models and selection of best model based on classical statistical parameters, Tropsha suggested that to simulate the use of QSAR models, it should consist of compounds with known activities/properties that are not included in either training or test sets. Even it was proposed that external evaluation set can be selected randomly from the entire initial dataset. In general, the size of the external evaluation set should be about 15%–20% of the entire dataset, and the remaining part of the dataset is called modeling set which can be split into the training and test sets. Since in some QSAR/QSPR works, the initial dataset do not consist of a large number of compounds so as to have the external evaluation set, in this work, we used an external data set published in different literatures. Performing this workflow is not only validated that the model is applicable for subsequent inhibitors and prediction purpose. Therefore, some new compounds with the similar core were used to develop the external evaluation set, and then, the statistical parameters for this set were calculated. The chemical structures and experimental data used with their prediction results are shown in Table 5. As it can be seen, the results are in good agreement for compounds E1-E5; however, to better understand the deviations between predicted and observed values, Euclidean based applicability domain was used to detect the outliers (41), Euclidean based applicability domain helps to ensure that the training set compounds employed in model development is representative for the compounds of the evaluation set. This method is based on the distance scores calculated by Euclidean distance norms. Firstly, the normalized mean distance score for training set compounds are calculated (these values ranges from 0 to 1 where 0.0 is least diverse, and 1.0 is the most diverse training set compound). Then, the normalized mean distance score for the test set compounds are calculated, and those test compounds which are scored outside of 0.0 to 1.0 ranges are defined to be outside of the applicability domain. The Euclidean based applicability domain is shown in Figure 5. Therefore, the reason behind these prediction results is the lower diversity of chemical structures comparing to the training set. In this respect, to predict the activities/properties of new compounds, it is here suggested to perform the Euclidean based applicability domain before performing any computing so as to be confident that the prediction of inhibition activity of compounds are within the capability of the models. One may refer to residuals values to compare the prediction results at once, but the presence of outliers can be normally detected by William plots as discussed above. 

The Williams plot for these five compounds was calculated and the results showed no presence of outliers. The Williams plot for molecules E1-E5 was shown in Figure 6.

Golbraikh and Tropsha acceptable model criteriaʹs are described as follows:

I) Q^2^_LOO_ value must be higher than 0.5.

II) R^2^ value must be higher than 0.6.

III) R_0_^2 ^_ R_0_^′2 ^/ R^2 ^< 0.1 and 0.85 < *K′ < *1.15 

or R^2 ^_ R_0_^2 ^/ R^2 ^< 0.1 or 0.85 < *K < *1.15

IV) R^2 ^_ R_0_^′2 ^< 0.3

Where R^2^ is correlation coefficient between the predicted and observed values; R_0_^2^ is coefficients of determination (correlation of predicted versus observed values with intercept of zero), and R_0_^′2^ is correlation between observed versus predicted values for regressions through the origin; K is slope and K′ is slope of regression lines through the origin. The results of these calculations for GA-MLR are listed in Table 6.

The final analysis to ensure that the model is established well and the molecular descriptors are selected appropriately is to derive a different set of molecular descriptors combinations by GA. The results of this analysis are listed in Table 7. Since the models are verified by test set and also above methodologies, the proposed models can be used to estimate the inhibition activities of new compounds within the applicability domain of the models. The series of novel compounds were drawn and then by using the GA-MLR model, which is simple and initially verified, the inhibition activities were obtained. The structures and the activities of the new designed compounds are shown in Table 8.

## Conclusions

In this study, MLR and SVM were used to develop the linear and nonlinear QSAR models for prediction of the inhibition activities of 17β-HSD3 inhibitors. The proposed models clearly demonstrated a good correlation between the chemical structures and inhibition activities of the studied compounds. The validation of the models using the leave one out cross-validation, external test set, and Y-randomization, Golbraikh and Tropsha acceptable model criteria’s, modified R^2^ values, concordance correlation coefficient values, Euclidean based applicability domain, and employing an external evaluation set showed that the proposed models have a good internal and external predictive power. Comparison between GA-MLR and GA-SVM methods demonstrated that the performance of GA-SVM model is better than that of GA-MLR suggesting that the nonlinear model is able to describe the relationship between the structural descriptors and the activity more accurately. The proposed models can identify and provide some insights about the chemical structural features required to derive inhibitors with high potency and thus, some instructions for successful synthesis of the potent 17β-HSD3 inhibitors.
